# Comparative transcriptome analyses shed light on carotenoid production and plastid development in melon fruit

**DOI:** 10.1038/s41438-021-00547-6

**Published:** 2021-05-01

**Authors:** Noam Chayut, Hui Yuan, Yuval Saar, Yi Zheng, Tianhu Sun, Xuesong Zhou, Anna Hermanns, Elad Oren, Adi Faigenboim, Maixia Hui, Zhangjun Fei, Michael Mazourek, Joseph Burger, Yaakov Tadmor, Li Li

**Affiliations:** 1 Department of Vegetable Research, ARO, Newe Ya’ar Research Center, P.O. Box 1021, Ramat Yishay, 30095 Israel; 2 Robert W. Holley Center for Agriculture and Health, USDA-Agricultural Research Service, Cornell University, Ithaca, NY 14853 USA; 3 Plant Breeding and Genetics Section, School of Integrative Plant Science, Cornell University, Ithaca, NY 14853 USA; 4 Boyce Thompson Institute, Ithaca, NY 14853 USA; 5 State Key Laboratory of Crop Genetics & Germplasm Enhancement, Nanjing Agricultural University, Nanjing, 210095 China; 6 Present Address: John Innes Centre, Norwich Research Park, Norwich, UK

**Keywords:** Comparative genomics, Transcriptomics

## Abstract

Carotenoids, such as β-carotene, accumulate in chromoplasts of various fleshy fruits, awarding them with colors, aromas, and nutrients. The *Orange* (*CmOr*) gene controls β-carotene accumulation in melon fruit by posttranslationally enhancing carotenogenesis and repressing β-carotene turnover in chromoplasts. Carotenoid isomerase (CRTISO) isomerizes yellow prolycopene into red lycopene, a prerequisite for further metabolism into β-carotene. We comparatively analyzed the developing fruit transcriptomes of orange-colored melon and its two isogenic EMS-induced mutants, *low-β* (*Cmor*) and *yofi* (*Cmcrtiso*). The *Cmor* mutation in *low-β* caused a major transcriptomic change in the mature fruit. In contrast, the *Cmcrtiso* mutation in *yofi* significantly changed the transcriptome only in early fruit developmental stages. These findings indicate that melon fruit transcriptome is primarily altered by changes in carotenoid metabolic flux and plastid conversion, but minimally by carotenoid composition in the ripe fruit. Clustering of the differentially expressed genes into functional groups revealed an association between fruit carotenoid metabolic flux with the maintenance of the photosynthetic apparatus in fruit chloroplasts. Moreover, large numbers of thylakoid localized photosynthetic genes were differentially expressed in *low-β*. CmOR family proteins were found to physically interact with light-harvesting chlorophyll a–b binding proteins, suggesting a new role of CmOR for chloroplast maintenance in melon fruit. This study brings more insights into the cellular and metabolic processes associated with fruit carotenoid accumulation in melon fruit and reveals a new maintenance mechanism of the photosynthetic apparatus for plastid development.

## Introduction

Carotenoids fulfill diverse biological functions in plants^1–3^. They are essential for plants due to their roles in photosynthesis and photoprotection. In chloroplasts, carotenoids facilitate light absorption in the blue-green light spectrum and protect antenna complexes from photooxidation^4^. Carotenoids also serve as precursors for phytohormone strigolactone and abscisic acid biosynthesis^5,6^. In chromoplasts of fruits and flowers of numerous species, carotenoids serve as pigments to attract pollinators and seed dispersers^7,8^. In edible fleshy fruits such as watermelons, melons, and tomatoes, carotenoid volatile derivatives influence fruit aroma and flavor^9,10^. Moreover, carotenoids are critically important for human nutrition and health in providing provitamin A and antioxidants.

**Fig. 1:**
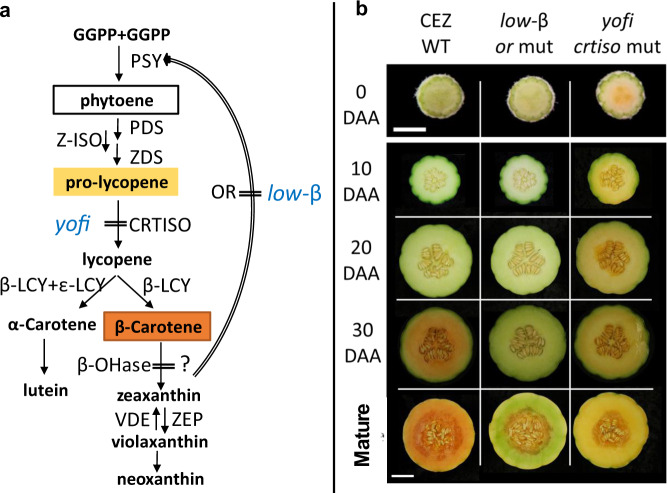
Schematic carotenoid biosynthetic pathway and melon fruit phenotypes. **a** Outline of carotenoid biosynthetic pathway. The carotenoids predominately accumulated in *yofi* and orange-flesh melon fruit are marked by their distinctive color. Vertical bar lines represent genetic inhibition imposed by loss-of-function mutation in the *yofi* and *low-β* mutants. OR posttranslationally regulates PSY activity. A question mark represents an unknown mechanism. GGPP geranylgeranyl diphosphate, PSY phytoene synthase, PDS phytoene desaturase, ZDS ζ-carotene desaturase, Z-ISO ζ-carotene isomerase, CRTISO carotenoid isomerase, β-LCY lycopene β-cyclase, ɛ-LCY lycopene ɛ-cyclase, β-OHase β-carotene hydroxylase, ZEP zeaxanthin epoxidase, VDE violaxanthin de-epoxidase. **b** Fruit phenotypes at different developmental stages of WT (CEZ), *low-β*, and *yofi* mutants. White bars on the bottom left of picture indicate 5 mm for ovaries (0 DAA) and 5 cm for developing fruits

Carotenoids are synthesized in plant plastids^11^. The first committed step in the carotenoid biosynthetic pathway is the condensation of two geranylgeranyl diphosphate molecules into phytoene by phytoene synthase (PSY) (Fig. 1a). PSY protein level and activity are regulated posttranslationally by the Orange protein (OR)^12^, a DnaJE1 family chaperone protein^3^. The non-colored *cis*-configured phytoene is desaturated and isomerized in a series of enzymatic reactions to yield 7,9,7′,9′-tetra-*cis*-lycopene (prolycopene). Carotenoid isomerase (CRTISO) converts the yellow colored prolycopene into the red colored all-*trans* lycopene^13,14^ (Fig. 1a). All-*trans* lycopene is the substrate for lycopene β- and ε-ring cyclases to yield β-carotene and α-carotene in the subsequent two branches of the metabolic pathway. Alpha-carotene is converted into lutein, the most abundant photosynthetic carotenoid, while β-carotene is hydroxylated to form various other xanthophylls^15^. Carotenes and xanthophylls can be further cleaved by carotenoid cleavage dioxygenase enzymes to produce various apocarotenoids including volatiles and scent molecules or phytohormones ABA and strigolactones^15^.

Carotenoid content and composition define the fruit mesocarp color of melon (*Cucumis melo*)^16^. The *CmOr* gene with a “golden” SNP as a dominant allele controls the accumulation of β-carotene, the predominant carotenoid in orange-fleshed melon fruit varieties^17,18^. *CmOr* was shown to possess two functional roles in melon fruit: it enhances carotenoid metabolic flux by posttranslational regulation of PSY1, and induces chromoplast formation with arrested β-carotene turnover in orange-fleshed fruit (Fig. 1a)^19^. PSY1 is similarly upregulated by both dominant and recessive allele products of *CmOr* gene, enabling similar carotenoid metabolic flux in developing fruit irrespective to final β-carotene accumulation^19,20^.

Mutant libraries with loss-of-function mutations in essential genes provide excellent genetic resources to investigate gene functions. An ethyl-methanesulfonate (EMS) mutagenesis library originating from an orange-fleshed “charentais” type melon line (CEZ) carrying dominant *CmOr* was visually screened for fruit quality phenotypes^21^. Two mutant lines displaying different fruit flesh color (named *low-β* and *yofi)* were identified and investigated^19,22^. The *low-β* mutant results from a mutation of *CmOr* containing an EMS-induced premature stop codon^19^. The loss of *CmOr* function leads to low β-carotene levels in mature fruit due to lower carotenoid metabolic flux caused by lower PSY1 protein level and absence of chromoplast formation. The *yofi* mutant is a mutation of *CmCRTISO* with an EMS-induced premature stop codon. It produces yellow-fleshed melon fruit due to high prolycopene accumulation. Prolycopene is accumulated in *yofi* fruitlets from the earliest stages of fruit development at the day of flowering^22^. The *crtiso* mutants were also identified in other crop species, such as *tangerine* (t) mutant of tomato^13^, watermelon of orange and salmon-yellow fruit flesh color^23^, and yellow Chinese cabbage^24^.

The global transcriptomes of green- and orange-fleshed melon fruit during fruit development were previously studied^18^. The *low-β* mutant represents the only *Or* impaired mutant in fruit crops. In this work, we investigated and compared the fruit transcriptomic changes caused by the loss of function of *CmOr* and *CmCRTISO* in comparison with their isogenic line CEZ. The major effect of *CmOr* on the fruit transcriptome was found in the ripe fruit, while *CmCRTISO* prominently affected at the early stages of fruit development. We associated each mutation with cellular and metabolic processes according to MapMan^25^ classification of the differentially expressed genes (DEGs), and searched for enriched processes and cellular component. We pointed out and discussed the effect of several specific genes that were differentially expressed in both mutants. In addition, a comparison of global transcriptome changes caused by *crtiso* in melon and Chinese cabbage^24^ suggests candidate genes involved in carotenoid associated processes in different organs and species. Furthermore, based on transcriptome analysis, we discovered a new role of CmOR in regulating light-harvesting chlorophyll a–b binding (LHCB) proteins to maintain photosynthetic apparatus and plastid development.

## Results and discussion

### Phenotypes of *low-β* and *yofi* mutant

The two studied mutants *low-β* and *yofi* carry recessive mutations identified from an EMS library^21^. The isogenic progenitor line for both mutants was CEZ, a climacteric cantaloupe melon of “charentais” type which is referred here as the wild type (WT). While the ripe WT fruit flesh was orange due to high levels of β-carotene and low levels of chlorophylls, the *low-β* mutant fruit flesh remained green throughout ripening, and the ripe *yofi* fruit flesh was yellow (Fig. 1b). At 20 days after anthesis (DAA), the fruit flesh color of *low-β* and of the WT was similarly light green. The color was notably different at 30 DAA with the *low-β* flesh remaining green and the WT fruit turning orange (Fig. 1b). In contrast, the *yofi* mutant phenotype arose early during fruit development, and the fruitlets were already yellow at 10 DAA when WT fruitlets were still light green (Fig. 1b).

The orange *Or* allele gene product stabilizes PSY and triggers chromoplast differentiation^3,11,26^. The *low-β* mutant is a *CmOr* nonsense mutation^19^. While the orange WT fruit accumulates ~30 μg/g fresh weight of β-carotene at mature stage, the *low-β* mutant contains 1 μg/g fresh weight^19^. The low β-carotene level in the *low-β* mutant fruit flesh results from low carotenoid metabolic flux due to reduced PSY protein level and defect in induction of chromoplast formation, which lead to few chromoplasts and more chloroplasts in the ripe fruit^19,20^. In the WT, the prolycopene produced is isomerized by CRTISO to yield all-*trans* lycopene, which is further metabolized to β-carotene in orange-flesh melon fruit. The *yofi* mutant is a loss-of-function mutation of *CmCRTISO*. The yellow color in the *yofi* mutant fruit flesh is caused by the accumulation of prolycopene due to the arrested carotenoid metabolic flux at the isomerization step^22^. It thus prevents the formation of all-*trans* lycopene and β-carotene in fruits (Fig. 1). Noticeably, *yofi* accumulates similar level of carotenoids as the orange WT fruit at mature stage^22^.

### Transcriptomes of *low-β* and *yofi* developing fruit

The *low-β* and *yofi* mutants represent the only two characterized mutants with mutations affecting carotenogenesis in melon fruit. To further investigate the *CmOr* and *CmCRTISO* gene functions and their influences on the associated metabolic and cellular processes, we examined the effects of each mutation on the global fruit transcriptomes and compared their impacts on transcription changes during fruit development.

**Fig. 2:**
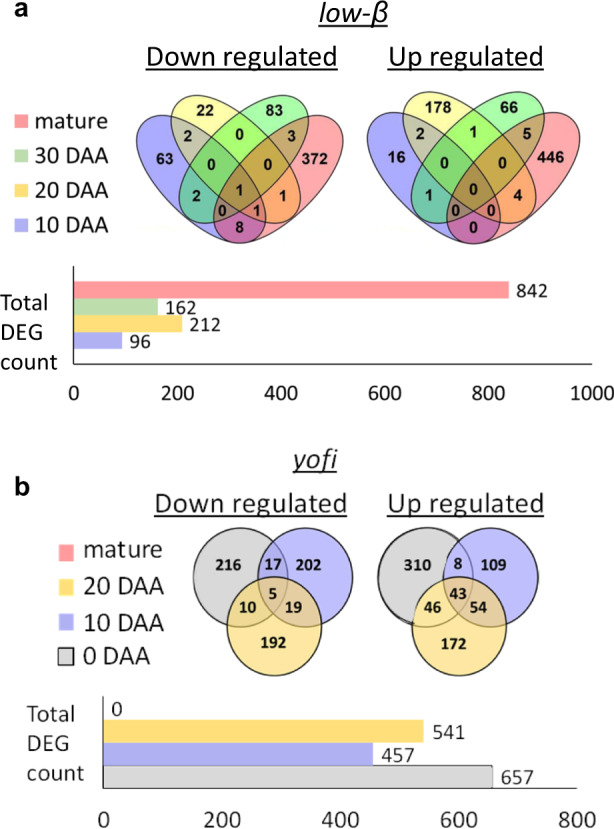
Count of differentially expressed genes at four fruit developmental stages of *low-β* and *yofi* mutants. DEG is a gene up- or downregulated at least twofolds in *low-β* (**a**) and in *yofi* (**b**) compared with WT with adjusted *p* < 0.05. The Venn’s diagrams show counts of DEGs uniquely down- or upregulated at each stage and the counts of DEGs mutually changed at different stages. Histograms show sum of DEG counts for each stage. DEG counts of fruitlets at 10 and 20 DAA and of ripe fruits are shown for both mutants. DEG counts of fruitlets at 30 DAA are shown only in *low-β* (**a**) and at the day of flowering (0 DAA) only in *yofi* (**b**)

To examine the influence of *CmOr* mutation on global transcription changes, we performed comparative RNA-Seq analysis between *low-β* and its isogenic progenitor WT at four fruit developmental stages: 10, 20, and 30 DAA and the mature fruit. We identified 96, 212, 162, and 842 DEGs at 10, 20, and 30 DAA and in the mature fruit, respectively (Fig. 2a, Table S1). Almost all the DEGs appeared uniquely in one developmental stage with only 29 DEGs (2.2%) up- or downregulated in more than one stage (Fig. 2a, Table S2). *CmOr* was the only DEG found in all the four examined stages: its expression was acutely lowered as a result of the mutation, most probably by a nonsense-mediated mRNA decay mechanism^27^.

A relatively low number of DEGs were found before fruit mature. However, the 96 DEGs observed at 10 DAA, a very early stage of fruit development prior to carotenoid accumulation and chromoplast formation, suggest that *CmOr* affects additional cellular processes. Notably, large number of DEGs was observed at the mature stage (Fig. 2a, Table S1). The major transcriptional changes at the mature stage in *low-β* likely arose as a result of the change in the carotenoid metabolic rate and chromoplast development due to the *CmOr* mutation^19^, along with the potential effects of *CmOr* on other cellular processes.

The *yofi* mutant is a *CmCRTISO* mutation that arrests carotenoid metabolism by preventing the formation of all-*trans* lycopene in the fruit (Fig. 1a). Since the mutant phenotype appeared at the early stages of fruit development and could even be observed at the day of flowering^22^, we sampled ovaries of open flowers (0 DAA) as the earliest analyzed time point. We further analyzed transcriptome changes in fruit at 10 and 20 DAA and mature stage. We identified 657, 457, and 541 DEGs at 0, 10, and 20 DAA, respectively (Fig. 2b, Table S3). Interestingly, we found no DEGs at the mature stage in spite of the dramatic difference in carotenoid composition with WT accumulating predominantly β-carotene and *yofi* mainly prolycopene^22^. In the four tested developmental stages, a total of 742 and 661 DEGs were up- and downregulated, respectively, in *yofi* as compared to WT. Out of the 742 upregulated DEGs, 151 (20.4%) were upregulated at more than one developmental stage, 43 of which were upregulated in 0, 10, and 20 DAA (Fig. 2b, Table S4). Out of the 661 DEGs that were downregulated in *yofi* at the sampled developmental stages, 51 (7.7%) were changed in more than one developmental stage (Fig. 2a, Table S4). *CmCRTISO* itself (*MELO3C009571*) was downregulated in the mutant in all developmental stages but exceeded the statistical cutoff only at 10 and 20 DAA (Table S3).

In mature *yofi* fruit, yellow prolycopene substitutes orange β-carotene of the WT progenitor (Fig. 1). However, no DEGs were observed at the mature stage despite the striking difference in color and in carotenoid composition. The surprising low change of transcriptome in the ripe fruit of *yofi* suggests that the substitution of β-carotene with prolycopene had a minimal effect on gene transcription at fruit mature stage. On a contrary, the high abundance of DEGs at the early fruit developmental stages indicates that the carotenoid metabolic flux arrest at prolycopene in *yofi* brings a significant change in fruit transcriptome during fruit development.

Mutations at *CRTISO* have been observed in some other plants and greatly affect various aspects of plant metabolism and development in addition to coloration in leaves and mature fruits^13,14,23,24,28,29^. In the *crtiso* mutants, prolycopene and low amounts of its upstream *cis*-carotenes are accumulated. The accumulation of these metabolites has been linked to processes such as regulation of chloroplast and nuclear gene expression, metabolic feedback regulation of pathway genes and proteins, and plastid development^28,30–33^. Prolycopene, upstream *cis*-carotenes, and their derivatives are hypothesized to exert their roles through acting as signal molecules^34^. The significant change in *yofi* fruit transcriptome during early fruit development but not at the mature stage implies greater responses of developing tissue to the accumulation of prolycopene or its related metabolites. The contrasting effects of pathway (*CmCRTISO*) and sequestration *(CmOr*) gene mutations on transcriptome changes at different fruit developmental stages in the *yofi* and *low-β* mutants show different roles of carotenoid metabolic processes in affecting global gene transcription.

### Cellular and metabolic processes associated with loss of function of *CmOr* and *CmCRTISO*

**Fig. 3:**
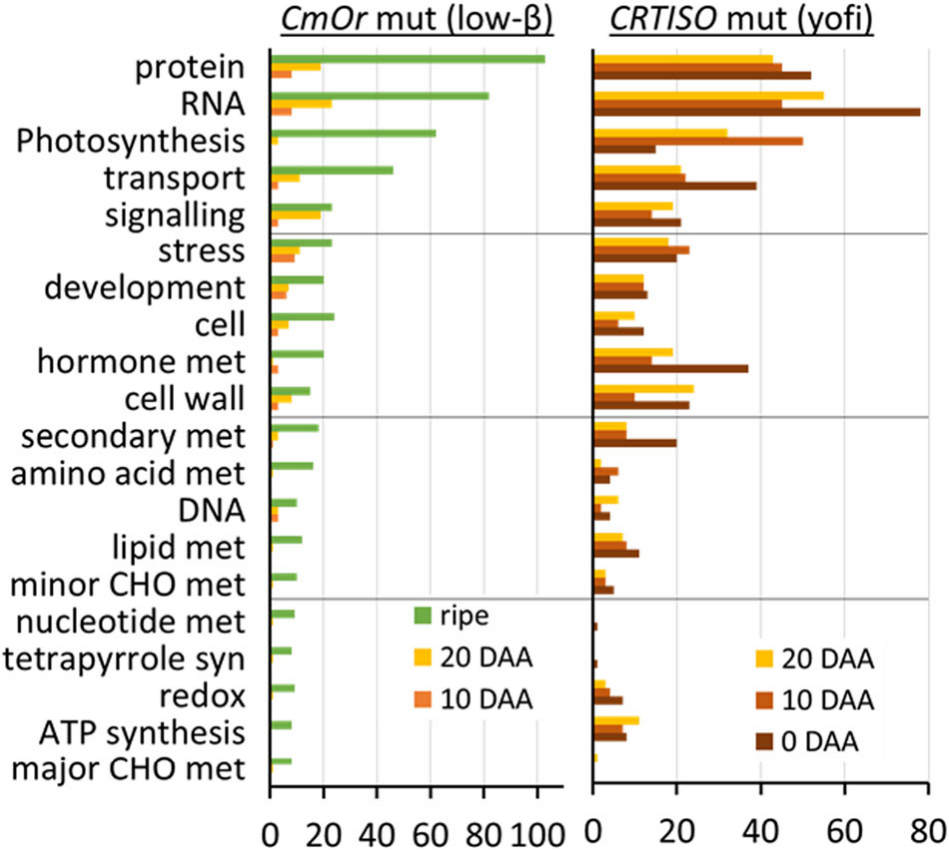
Cellular and metabolic processes affected by *Cmor* and *Cmcrtiso*. The DEGs at each developmental stage in *low-β* (left panel) and in *yofi* (right panel) were categorized according to the bin-codes of MapMan^25^. Counts of DEGs in each category are presented

We clustered the DEGs in *low-β* and in *yofi* as compared to the WT into functional groups using MapMan^25^. In *low-β* ripe fruit, the most abundant functional groups of DEGs were those involved in protein processing (103 DEGs), RNA regulation (82), and photosynthesis (62) (Fig. 3, Table S1). In *yofi*, on the day of flowering at 0 DAA, the most abundant functional groups were those involved in RNA regulation, protein processing, and transport (78, 52, and 39 DEGs, respectively). At 10 DAA, most DEGs were related to photosynthesis, RNA regulation, and protein processing (50, 45, and 45 DEGs, respectively), and at 20 DAA the abundant groups of DEGs were related to RNA regulation, protein processing, and photosynthesis (55, 43, and 32 DEGs, respectively) (Fig. 3, Table S3).

We sought to identify cellular and metabolic processes that were transcriptionally regulated by both mutations to affect carotenoid metabolism. We hypothesized that the mutual changes would point out specific genes and processes that are linked to carotenogenesis. The total numbers of DEGs altered by *yofi* and by *low-β* were 1331 and 1228, respectively. Overlapping these two groups returned 260 genes, which were categorized according to the functional hierarchy of MapMan (Fig. S1, Table S5). Among them, 188 DEGs could be categorized into various cellular and metabolic processes. Out of these genes, the most abundant cluster was associated with photosynthesis (27 DEGs). Other abundant groups were related to RNA and protein (26 and 23 DEGs, respectively) (Fig. S1, Table S5).

#### Carotenoid metabolic genes

The *low-β* and *yofi* mutants exhibit defects in carotenoid biosynthesis and accumulation. The carotenoid metabolic gene expressions in the two mutants were previously investigated^19,22^. The loss of *CmOr* function did not affect carotenoid biosynthetic pathway gene expression at most of fruit developmental stages except several genes showing upregulation at fruit mature stage in *low-β* (Table S1). The absence of correlation between carotenoid metabolic pathway gene expression and the altered carotenoid levels in *low-β* is consistent with previous studies, which show no differential expression in carotenoid metabolic genes with elevated carotenoid accumulation conferred by *Or* in cauliflower, *Arabidopsis*, and melon^18,35,36^.

In *Cmcrtiso* melon, carotenoid biosynthetic genes upstream of *CmCRTISO* were reported to be just slightly upregulated in ripe fruit^22^. Consistently, we found no genes in the carotenoid metabolic pathway that were significantly differentially expressed because of the *CmCRTISO* mutation (Table S3). The *crtiso* loss-of-function mutation in tomato was shown to upregulate *PSY1* transcription in a genetic background of yellow flesh (locus *r*), which reduces fruit carotenoids by disrupting the activity of PSY1^28^. The authors suggested that the regulation of *PSY1* gene expression is mediated by *cis*-carotenoid metabolites. In contrast to the findings in tomato, the melon *PSY1* (*MELO3C025102*) expression was not significantly mediated by the loss of *CmCRTISO* function. A significant change of 9-*cis*-epoxycarotenoid dioxygenase-1 (*NCED-1*; *MELO3C027057*) was observed in *yofi* at 0 DAA, which showed a 15.6 time upregulation (Table S3). NCED enzymes catalyze a key step in ABA biosynthesis, the cleavage of neoxanthin and violaxanthin into xanthoxin. *NCED-1* upregulation in *yofi* was presumably a result of less available substrate from the metabolic flux arrest imposed by the *CmCRTISO* mutation.

#### Photosynthesis related genes

In *low-β* ripe fruit, 61 DEGs were clustered to the functional group of photosynthesis by MapMan; all of them (100%) were upregulated in the mutant as compared to WT (Table S1). The subclassification of the photosynthetic upregulated genes in *low-β* included 24 DEGs of light-reaction photosystem II, 11 of light-reaction photosystem I, and 14 from Calvin cycle comprising the subunits of Rubisco and Rubisco interacting proteins (Table S1). We previously showed that during the first 30 days of fruit maturation, total chlorophyll contents in both WT and *low-β* increased similarly in the mesocarp, but a sharp decrease was observed in WT in contrast to remaining relatively high chlorophyll levels in *low-β* of the ripe fruit^19^. The upregulation of photosynthetic genes was consistent with the observed green-flesh phenotype in the *low-β* mutant (Fig. 1b).

In *yofi* fruitlets, 15, 50, and 32 genes were clustered to photosynthesis at 0, 10, and 20 DAA, respectively (Fig. 3, Table S3). Out of which, 14, 29, and 32 were respectively upregulated in the mutant as compared to the WT (Table S3). At 20 DAA, the subclassification included 30 DEGs of the light reaction with 9 related to ATP synthase, 6 to the cyclic electron flow of photorespiration, 7 to photosystem I, 4 to photosystem II, and 4 to cytochrome b6/f. The overlapping photosynthetic genes that were differentially expressed in both mutants included 27 genes, related to the light reaction in photosystem II (13) and photosystem I (8), to Calvin cycle (2), to electron flow (2), and other electron carrier (2) (Fig. S1, Table S5).

Transcriptome analysis of the effects of *CRTISO* mutation on global gene expression changes was also performed in the *crtiso* Chinese cabbage leaves. In Chinese cabbage, no photosynthetic genes were upregulated and only two chlorophyll a–b binding family protein related genes were found mildly downregulated^24^. This is likely due to that the sampled Chinese cabbage inner leaves were either white or yellow with arguable low photosynthetic capacity. The observed changes of photosynthetic genes in melon *crtiso* mutant may suggest that the upregulation of photosynthetic genes because of carotenoid flux arrest at prolycopene takes place during early fruit development.

In the ripe fruit, the WT accumulates high amounts of β-carotene as a result of low β-carotene turnover and chromoplast development, whereas *yofi* accumulates high levels of prolycopene as a result of malfunctioning CRTISO, and *low-β* accumulates low amounts of chloroplastic carotenoids because of the *CmOr* mutation^19,22^. The results obtained here showed that substitution of β-carotene with prolycopene in the ripe *yofi* fruit led to a minimal effect on transcription of plastid and photosynthetic related genes unlike at early fruit developmental stages. It appears that the transcriptional maintenance of the photosynthetic apparatus continues toward fruit ripening if carotenoids are not accumulated. This was concluded because numerous genes which encode essential structural photosynthetic elements maintained high expression levels in the ripe *low-β* fruit but were downregulated in WT (Table S1) and similarly downregulated in *yofi*.

#### RNA related genes

In *low-β*, a total of 8, 23, and 82 DEGs were clustered as RNA regulators at 10 and 20 DAA and the mature stage, respectively (Fig. 3, Table S1). Among the 82 DEGs in the ripe fruit, 68 DEGs were transcription factors (TFs), the rest were related to RNA processing (8), RNA binding (4), and RNA transcription (4). Out of the 68 differentially expressed TFs, 29 were upregulated and 39 were downregulated in *low-β* mutant as compared to the WT. These TFs belonged to several families, the most abundant ones were AP2/EREBP (7), Constans-like zinc finger, C2H2 zinc finger, and GOLDEN2-like (5 each) (Table S1).

In the *yofi* mutant, 78, 45, and 55 genes were clustered as RNA regulators at 0, 10, and 20 DAA, respectively (Fig. 3, Table S3). At 0 DAA, 75 out 78 DEGs were TFs and 3 DEGs were related to RNA processing. Out of the 75 differently expressed TFs, 67 were upregulated and 18 were downregulated in *yofi* as compared to WT. The main subfamilies of these TFs were MYB (12), AP2 /EREBP (9), bHLH (8), and WRKY (5).

Among the DEGs that were affected by the two mutations throughout fruit development in *low-β* and *yofi*, 26 DEGs were TFs abundant from the families of AP2/EREBP, C2H2 zinc finger, and WRKY (Fig. S1, Table S5). In recent years, TFs that specifically control transcription of carotenoid metabolism genes have been identified^3,37^. Nevertheless, none is known to specifically regulate or bind to the promoters of *Or* and *CRTISO* in activating/suppressing their expression in fruits. OR has been shown to physically interact with TF TCP14 to repress TCP14 transcriptional activity in etiolated *Arabidopsis* cotyledons^38^. The *CRTISO* expression was found to require a chromatin-modifying histone methyltransferase enzyme (SDG8), an epigenetic regulatory mechanism in *Arabidopsis*^39^. Those TFs commonly affected by both gene mutations in *low-β* and *yofi* might play a role in regulating *Or* and *CRTISO* expression or be associated with altered carotenoid metabolism, which warrant further study.

#### Protein processing related genes

In *low-β*, a total of 8, 19, and 103 DEGs were related to protein processing at 10 and 20 DAA and the mature stage, respectively (Fig. 3, Table S1). Protein processing was the largest cluster of DEGs in *low-β* ripe fruit and was subdivided as following: protein degradation (47), posttranscriptional modification (21), protein synthesis (20), protein targeting (8), protein folding (4), and protein assembly (2). Among the 20 DEGs related to protein synthesis, 15 were ribosomal subunits of the chloroplast. Although nearly equal numbers of protein related DEGs were up- or downregulated in the protein processing classification, all the 15 chloroplastic ribosomal genes were upregulated in *low-β* (Table S1).

In *yofi*, a total of 52, 45, and 43 DEGs were related to protein processing at 0, 10, and 20 DAA fruitlets, respectively (Fig. 3, Table S3). The protein processing related DEGs in *yofi* fruitlets were subdivided as following: protein degradation (50), posttranscriptional modification (29), protein synthesis (20), protein targeting (4), protein folding (3), and protein assembly (3). Among the 20 DEGs related to protein synthesis, 11 were ribosomal subunits of chloroplasts and they were all upregulated in *yofi* (Table S3). The upregulation of chloroplastic ribosomal genes in both *low-β* and *yofi* mutants is most probably another indication of the effect of the mutants on plastid fate.

In the overlapping group of DEGs that were differentially expressed in both mutants, 23 were related to protein processing (Fig. S1, Table S5). They were divided into three subgroups: protein degradation (15), posttranscriptional modification (6), and protein synthesis (6) (Table S5). The six DEGs related to protein synthesis were ribosomal subunits of chloroplasts.

Therefore, in accordance with the aforementioned photosynthesis related DEG cluster, it appears that transcriptional maintenance of the photosynthetic apparatus continues toward fruit ripening as long as carotenoid accumulation does not occur. The protein processing related DEGs were also abundantly related to changes in plastid activity. The finding further suggests that transcriptional maintenance of chloroplasts is active in the ripe fruit of *low-β* as opposed to the WT and *yofi*. This could be concluded by the high expression of chloroplast ribosomal proteins in *low-β* in contrast to WT mature fruit and *yofi* developing fruits. The high carotenoid accumulation in WT ripe fruit might cause transcriptional changes that lead to plastid conversion and chloroplast degradation. Indeed, a recent study reveals that a stimulation of carotenoid biosynthesis along with the loss of photosynthetic competence initiates a major reprogramming of global gene expression to prompt the differentiation of chromoplasts from chloroplasts^40^.

### Highlight of specific DEGs in both *CmCRTISO* and *CmOr* mutated melon fruit

*MELO3C024423* encodes a leucine-rich repeat protein kinase (LRRPK) and was differentially expressed in both mutants (Table S5). At 20 DAA, it was downregulated by fivefold in *yofi* and upregulated by 5.6-fold in *low-β* as compared to WT. Thus, in both melon mutants, this LRRPK gene expression was strongly inversely correlated to the *CmCRTISO* and *CmOr* gene mutations, suggesting that MELO3C024423 has a role related to the change in melon fruit carotenoid metabolism. Its *Arabidopsis* ortholog (IOS1; At1g51800) is involved in negative regulation of ABA-activated signaling pathway^41^. This excites a hypothesis that MELO3C024423 LRRPK possibly adjusts ABA sensitivity to ABA production in fruits.

*MELO3C026802* encodes a magnesium-protoporphyrin monomethyl ester cyclase involved in chlorophyll synthesis. It was differentially expressed in both mutants in inverse correlation to the *CmCRTISO* and *CmOr* gene mutations, showing a 2.1-fold downregulation in *yofi* at 10 DAA and a 4.9-fold upregulation in ripe *low-β* fruit as compared to WT (Tables S1 and S3). Its *Arabidopsis* ortholog (AT3G56940) functions as a DNA-binding protein and is associated to chlorophyll biosynthetic process in chloroplast^42^. A second DEG *MELO3C016714* related to tetrapyrrole synthesis was changed in both mutants in a similar pattern with *MELO3C026802*, with a 2.1- and 4.3-fold downregulation in *yofi* at 10 and 20 DAA, respectively, and 10.4-fold upregulation in ripe *low-β* (Tables S1 and S3). The differential expression of a tetrapyrrole synthesis related gene further supports the conclusions deduced from the photosynthesis and protein processing related DEGs: the carotenoid accumulation followed by carotenoid pathway flux arrest retards the transcriptional maintenance of the chloroplast photosynthetic apparatus.

### Highlight of specific DEGs in *CRTISO* mutated melon fruit and Chinese cabbage leaves

The specific effects of the loss of *Or* function on global transcriptome have not been assessed in another plant species prior to this work. However, an analysis of *crtiso* mutated Chinese cabbage leaves was available, in which 372 DEGs were identified^24^. Based on sequence homologies, we found 342 corresponding genes in the melon genome. Among these 342 homologs, we searched for genes that were differentially expressed in at least one of the studied developmental stages. We found 41 genes which are listed in Table S6. Two hormone metabolism related genes were differentially expressed in cabbage leaves and in developing melon fruits, both were related to ethylene synthesis. ACC oxidase was downregulated in cabbage (*Bra031798*; 16.7-fold change) and in 20 DAA melon fruit (*MELO3C007425*; 2.1-fold change) (Table S6). Lowering the expression of ACC oxidase delays fruit ripening in melon^43^ and delays leaf senescence in tomato^44^. This result reinforces the suggested regulatory link between carotenoid metabolism and ripening.

Two TFs were found to be downregulated in both Chinese cabbage and melon species: an ethylene-responsive TF ERF113 (*Bra006195*, 2.5-folds and *MELO3C022358*, 4.3-folds at 10 DAA) and WRKY DNA-binding protein 70 (*Bra014692*, 7.7-folds and *MELO3C009097*, 2.9-folds at 20 DAA) (Table S6). Myo-inositol-1-phosphate synthase (*MIPS1*) was upregulated in both plant systems (*Bra011830*; 2.94-folds and *MELO3C017581*; 5.22-folds in 20 DAA). MIPS catalyzes the first committed step in the synthesis of inositol-containing compounds such as phospholipids, converting D-glucose 6-phosphate into 1L-myo-inositol-1-phosphate. MIPS overexpression in *Arabidopsis* resulted in higher carotenoid and chlorophyll levels by protecting the photosystem II^45^. Our results showed that this established metabolic link may also be reversed because the arrest in carotenoid metabolism induced by *crtiso* in *yofi* melon and in Chinese cabbage^24^ upregulated MIPS expression.

Interestingly, a gene encoding the chloroplast signal recognition particle component cpSRP43 was downregulated in both species (*Bra014106*, 0.47-folds and *MELO3C013306*, 0.37-folds at 10 DAA). cpSRP43 is known as a targeting factor for the thylakoid membrane, and as a chaperone with high specificity to the light-harvesting chlorophyll a–b binding proteins^46^. Other genes differentially expressed in the distinct organs of the two species included five transport related genes, two cytochrome P450s, two glutathione S transferases, three signaling related genes, three stress related, and two lipid metabolism related genes. Taken together, the common DEGs may suggest several conserved associations between carotenoid metabolic arrest and cellular and metabolic processes. These network associations would need to be further validated in other species or in the same species in future.

### The *low-β* induced DEGs are enriched in the thylakoid membrane

**Fig. 4:**
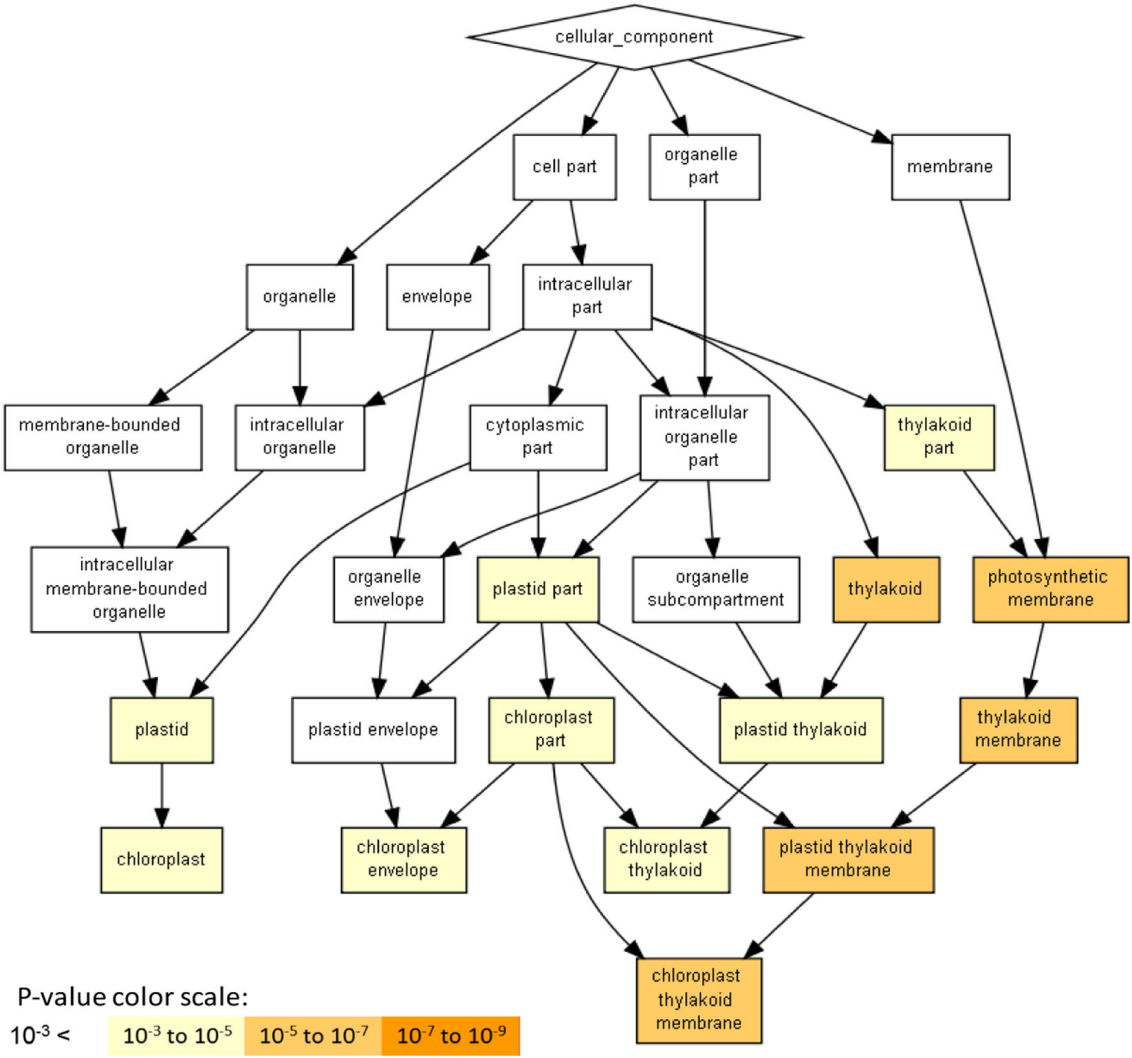
Cellular components enriched for the DEGs of *low-β* at the mature stage. *Arabidopsis* homologs of DEGs from ripe fruit of *low-β* were given as input to Gorilla. The resulting enriched GO terms are visualized using a DAG graphical representation with color coding reflecting their degree of enrichment. Enrichment is defined as (*b*/*n*)/(*B*/*N*); *N* is the total number of genes; *B* is the total number of genes associated with a specific GO term; *n* is the automatically determined number of genes in the “target set,” and *b* is the number of genes in the “target set” that are associated with a specific GO term. The annotated gene list of the enriched categories is listed in Table S7

As many DEGs were found in the ripe fruit of *low-β*, we sought to determine the cellular components mostly influenced by *CmOr* mutation at this fruit developmental stage. The *Arabidopsis* homologs of the DEGs (522 genes) at the ripe stage of *low-β* vs WT were analyzed using Gorilla (http://cbl-gorilla.cs.technion.ac.il/), a gene ontology enrichment analysis and visualization tool^47^. The enriched cellular compartments included several plastid-related subplastidial components including chloroplast envelope and chloroplast thylakoid. The genes localized in plastid thylakoid and thylakoid membrane were the most significantly enriched (Fig. 4, Table S7).

OR protein was found to directly interact with PSY and posttranslationally upregulate it in melon as well as in a number of plant species^12,19,48–51^. OR regulates PSY activity, which in turn controls the biosynthesis of carotenoid pigments necessary for both chloroplast and chromoplast development^12,52^. In addition, OR regulates chromoplast division to affect chromoplast number^53^. OR protein from sweet potato was found to physically interact with the oxygen-evolving enhancer protein 2-1 and therefore directly stabilize photosystem II in the thylakoid membrane^54^. OR was recently classified as a DnaJE1 family protein and several members of the DnaJE1 family are shown to participate in photosynthetic protein assembly^55^. The fact that the DEGs in the plastid thylakoid and thylakoid membrane were significantly enriched with the mutation of the *CmOr* gene suggests a potential role of *CmOr* in regulating processes in thylakoids in addition to its known functions.

### RT-qPCR confirms upregulation of genes encoding chlorophyll a–b binding proteins in *low-β* mature fruit

Photosystems locate in thylakoid membranes of the chloroplasts in plants. In *low-β* ripe fruit, we found 24 DEGs annotated as related to the light-reaction photosystem II with 13 of them encoding LHCB proteins (Table S1). The upregulation of 12 of these LHCB genes as well as the expression of *CmOr* and *CmOr-like*, *PSY1*, and *PSY2* with reads per kilobase of exon model per million mapped reads (RPKM) values from the RNA-Seq analysis in the *low-β* ripe fruit is shown in Fig. S2a (top panel). We also examined their expressions in *low-β* vs WT ripe fruit by RT-qPCR. Consistent with results from RNA-Seq analysis, all the LHCB protein genes showed upregulation in mature fruit of *low-β* compared to WT (Fig. S2a, low panel). Similar patterns of alterations with *Or* and *PSY* genes were also observed. The correlation between the two methods is presented (Fig. S2b).

LHCB proteins bind to chlorophylls and carotenoids for light harvesting and are critical for photosynthesis and chloroplast function^56^. Regulation of their expression is considered as an important mechanism for plants to mediate chloroplast functions. It is known that multiple environmental and developmental signals regulate *LHCB* gene expression, such as chloroplast retrograde signals, light, and phytohormone ABA^57–59^. Therefore, many upregulated *LHCB* genes in the *low-β* ripe fruit could indicate that the impairment in chloroplast to chromoplast conversion serves as another mechanism to affect the expressions of *LHCB* genes.

### CmOr and CmOr-like physically interact with chlorophyll a–b binding proteins to affect plastid development

The genes localized in plastid thylakoid membrane were most significantly enriched in the ripe fruit of *low-β* (Fig. 4). A large number of DEGs encoding LHCB proteins were among them (Table S1). Since LHCB proteins are important for chloroplast development and OR has a known role in physically interacting with oxygen-evolving enhancer protein 2-1 (PsbP) to stabilize the photosystem II protein in the thylakoid membrane^54,60^, we hypothesized that OR protein might also directly interact with LHCB proteins to potentially affect chloroplast development. To test it, we performed protein–protein interaction analysis using bimolecular fluorescence complementation (BiFC) assay and yeast two-hybrid (Y2H) assay. LHCB proteins belong to a large family that is classified as LHCB1, LHCB2, LHCB3, LHCB4, LHCB5, and LHCB6 in *Arabidopsis*^56^. Among the various subgroups of LHCB proteins, LHCB1 proteins are the most abundant contributors to light harvesting^56^. Thus, two LHCB1 protein genes (*MELO3C007154* and *MELO3C012912*), which are homologs of *Arabidopsis* *LHCB1.1* and *LHCB1.5*, respectively, and showed high differential expression between *low-β* and WT (Fig. S2a), were selected for protein–protein interaction analysis. In addition, since CmOR (MELO3C005449) and CmOR-like (MELO3C024554) are family proteins and both of them interact with PSY^12^, we also tested the interactions of CmOR-like with these two LHCB1 proteins.

**Fig. 5:**
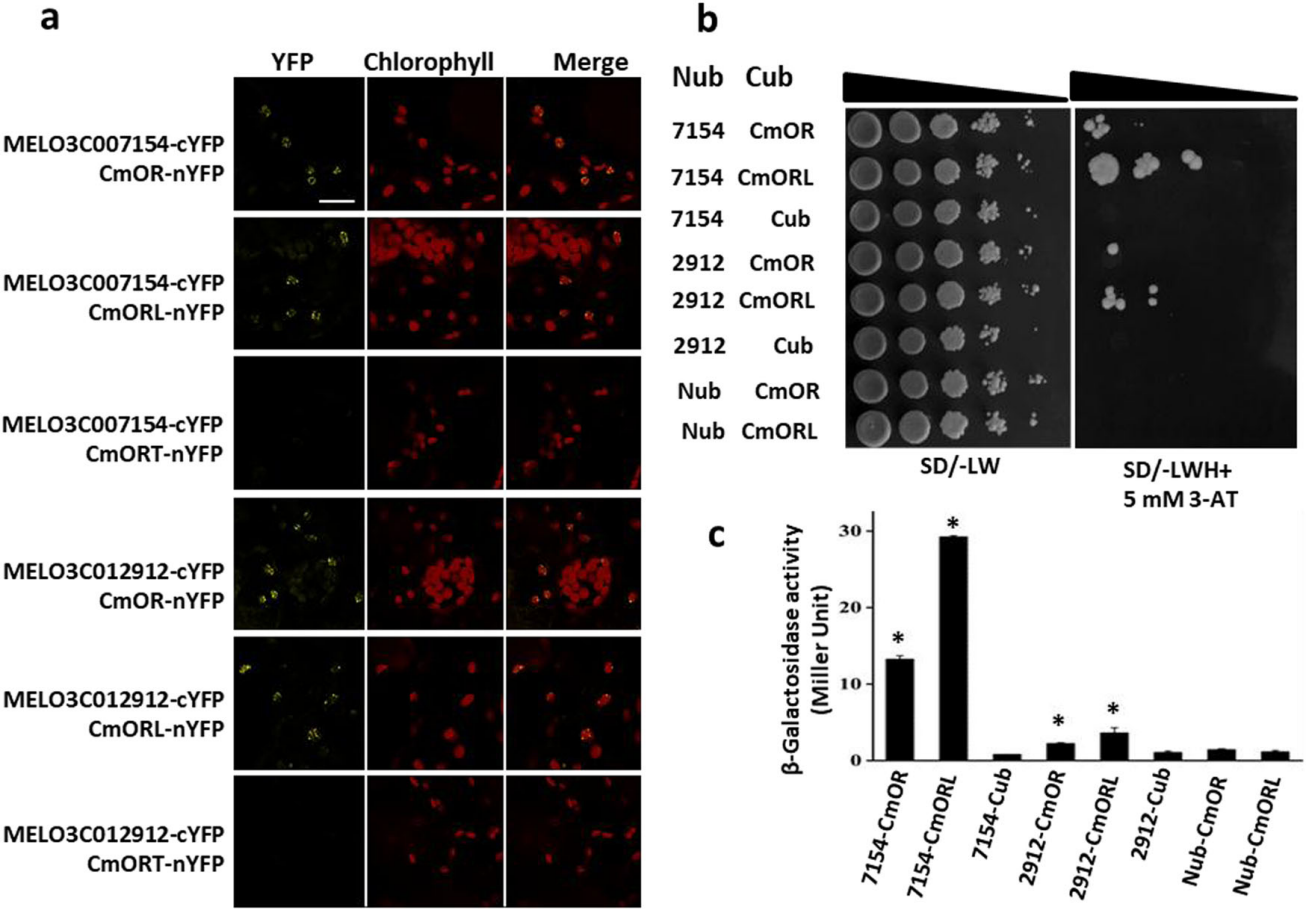
Interactions of CmOR and CmOR-like with two LHCB proteins. **a** BiFC analysis. CmOR (MELO3C005449), CmOR-like (CmORL, MELO3C024554), or CmORT (truncated form of CmOR from *low-β*) as an N-terminal fusion of YFP, and LHCB protein (MELO3C007154 or MELO3C012912) as a C-terminal YFP fusion of YFP were coexpressed in *N. benthamiana* leaves. YFP signals were observed by confocal microscope. Bar equals 20 µm. **b** Y2H assay. Yeast cotransformed with the constructs or empty vectors (Nub or Cub) as indicated was spotted with a series of tenfold dilutions on no-selection SD/-LW medium and selection SD/-LWH medium with 5 mM 3-AT. 7154, MELO3C007154; 2912, MELO3C012912. **c** Quantification of interaction strength by β-galactosidase activity assay. oNPG (ortho-nitrophenyl-β-galactoside) was used as substrate. Results are means + SD from three biological replicates. The asterisks indicate significant difference in comparison with controls (*p* < 0.05)

As shown in Fig. 5a, when the fusion proteins with N-terminal half of YFP fused to CmOR or CmOR-like and the C-terminal half of YFP fused to MELO3C007154 (CmLHCB1.1) or MELO3C012912 (CmLHCB1.5) were coexpressed in tobacco leaves, YFP signals were clearly observed, showing that both CmOR and CmOR-like physically interact with these two LHCB proteins. Noticeably, the truncated CmOR from *low-β* lost the ability to interact with these two LHCB proteins. No YFP signals were detected in the negative controls when vector only constructs were coinjected into tobacco leaves (Fig. S3). The interactions between CmOR or CmOR-like and CmLHCB1 occurred in chloroplasts (Fig. 5a), consistent with the thylakoid localization of LHCB1.

To further confirm the interactions, we performed Y2H analysis. When yeast was cotransformed with CmOR or CmOR-like and MELO3C007154 (CmLHCB1.1), they grew in the SD/-LWH selection medium with 5 mM 3-amino-1,2,4-triazole (3-AT) that suppresses the background growth of yeast (Fig. 5b). Similarly, when the yeast was cotransformed with CmOR or CmOR-like and MELO3C012912 (CmLHCB1.5), they also grew in the selection medium. In contrast, the controls with empty vectors did not grow in the selection medium. These results confirm the direct interactions between CmOR or CmOR-like with these two LHCB proteins. Their interaction strength was also quantified using the β-galactosidase activity assay (Fig. 5c). CmOR-like had stronger interaction strength with MELO3C007154 (CmLHCB1.1) or MELO3C012912 (CmLHCB1.5) than CmOR. The strongest interaction strength was observed between CmOR-like and MELO3C007154 (CmLHCB1.1), consisting with the yeast growth on the selection medium as shown in Fig. 5b.

**Fig. 6:**
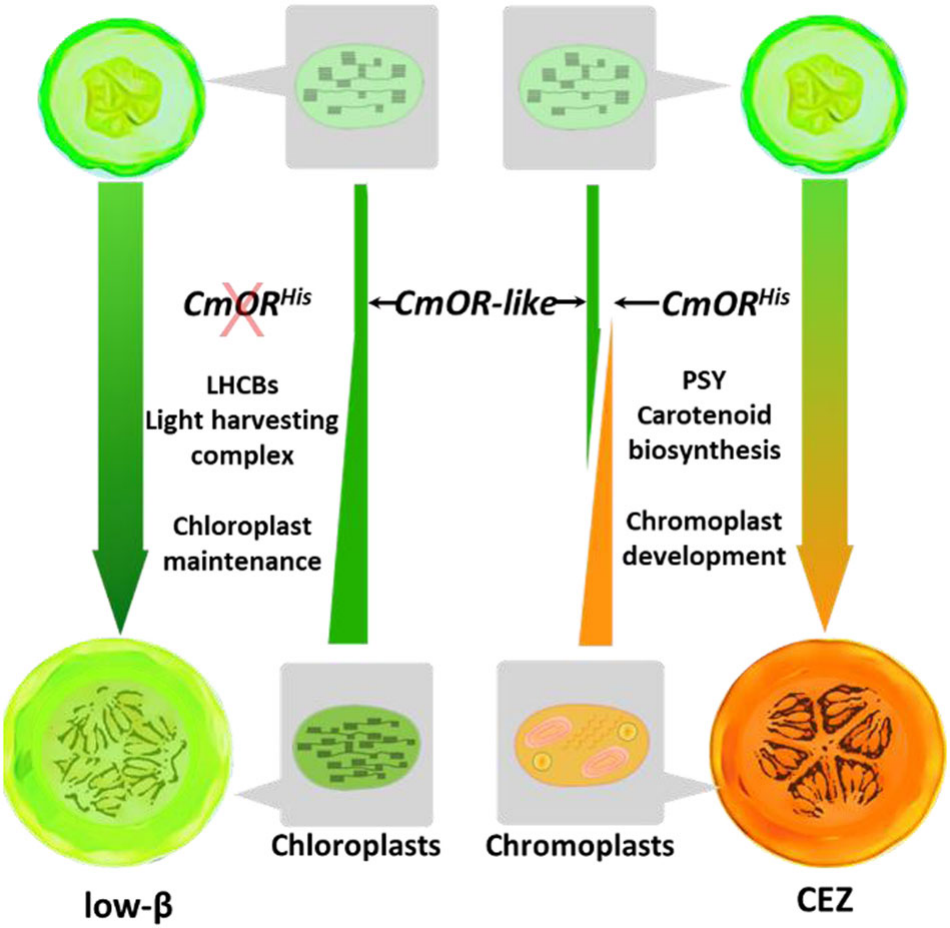
Model of distinct carotenoid accumulation and plastid development controlled by CmOR in CEZ and *low-β* fruit. During CEZ fruit ripening, CmOR induces chromoplast development and facilitates carotenoid biosynthesis through interaction with PSY, which result in the transition of chloroplasts to chromoplasts and the change of color from green to orange with carotenoid accumulation. In *low-β* fruit, the loss of function in CmOR cannot induce chromoplast formation and carotenoid accumulation during fruit ripening. Because of the functional redundancy of CmOR and CmOR-like in interacting with LHCB proteins, the chloroplast development is maintained to produce green-flesh mature fruit

In the WT melon fruit, ripening is accompanied with plastid transition from chloroplasts to chromoplasts and change of color from green to orange, whereas the *low-β* mature fruit maintains chloroplasts with green-flesh phenotype^19^. At the ripe stage, a predominant role of CmOR is to promote the conversion of chloroplasts into chromoplasts following the increased production of carotenoids. During early fruit development, CmOR may have a mechanism to help maintain chloroplast formation. Indeed, OR as a DnaJ-like cochaperone has been shown to maintain PSY and PsbP protein stability in green tissues via physical interaction with these proteins to promote photosynthetic carotenoid biosynthesis and protect photosystem II^11,53^. Here, we documented that both CmOR and CmOR-like physically interact with the LHCB proteins in vitro and in vivo. The direct interactions may affect LHCB protein stability, supporting the maintenance of the photosynthetic apparatus and chloroplast development. Although loss of the CmOR function in *low-β* diminished its ability to physically interact with LHCB proteins, the functional redundancy of CmOR and CmOR-like in regulating LHCB1 proteins enabled CmOR-like in *low-β* to main chloroplast development. The activity of CmOR-like protein explains the maintenance of chloroplast development in *low-β* with green-flesh phenotype. A model of CmOR along with CmOR-like in affecting carotenoid biosynthesis and plastid conversion during fruit ripening in WT and the *low-β* is presented in Fig. 6.

## Conclusion

Transcriptome analysis is a useful tool to associate specific gene function with global metabolic and cellular processes and to raise new hypotheses about the genetic network. In this study, we analyzed transcriptomes of a climacteric orange-fleshed melon and two independent isogenic lines baring mutations that halt carotenoid metabolic flux and affect chromoplast differentiation. The analysis suggested that fruit developmental transcriptome is greatly altered by changes in carotenoid metabolic flux rate but minimally by the carotenoid composition at the mature stage. The major transcriptional changes resulting from alteration of carotenoid metabolic rate also indicate the importance of maintaining carotenoid levels due to the interactions of carotenoids with many metabolic pathways and cellular processes. Moreover, if carotenoids do not accumulate, the transcriptional maintenance of the photosynthetic apparatus continues in the chloroplasts toward fruit ripening, suggesting a major role for carotenoid accumulation in triggering chloroplast to chromoplast transition in melon fruit. This is supported by a recent study that the differentiation of chromoplasts from chloroplasts necessitates an enhanced carotenoid production along with a reduced photosynthetic activity^40^. The enrichment of photosynthesis related DEGs with many of which localized in thylakoids in *low-β* mutant facilitated spotting a new role of CmOR family proteins in interacting with LHCB proteins to maintain chloroplast development.

## Material and methods

### Plant materials

The WT line used here was CEZ, a “charentais” type orange-flesh melon (*Cucumis melo* L. subsp*. melo* var*. cantalupensis* Naudin). The *low-β* and *yofi* mutant lines are EMS mutated CEZ isogenic lines, which were identified from M_2_ families of CEZ based EMS library^21^. The WT, *low-β*, and *yofi* melon plants were grown under standard horticultural conditions in the field of Newe Ya’ar Research Center, northern Israel. Female flowers were tagged at the day of anthesis. Fruit were sampled at 10 and 20 DAA and at mature stage (40–45 days varied among fruit). CEZ and *yofi* were additionally sampled at flowering day, whereas CEZ and *low-β* were additionally sampled at 30 DAA. Each biological replicate contained fruit from a different plant. Representative portions of the fruit mesocarp tissues were frozen in liquid nitrogen and kept at −80 °C until use.

### RNA-Seq transcriptome analysis

Total RNA and RNA-Seq library preparation were conducted as previously described^19^. The extracted total RNA was quantitated using a NanoDrop spectrophotometer. The RNA integrity was assessed with an Agilent Bioanalyzer. A total of 50 μg RNA from each sample were utilized to construct the strand-specific RNA-Seq libraries, which were sequenced on an Illumina HiSeq 2000 sequencer at the Cornell University core facility (http://www.brc.cornell.edu/brcinfo/) for *low-β* and CEZ and at the Technion infrastructure unit (https://isu.technion.ac.il/) for *yofi* and CEZ. The experiments on *low-β* and *yofi* were performed with three and two biological repeats, respectively, and both were with four developmental stages. An average of about 7.4 million reads from each library was produced.

### RNA-Seq data analysis

RNA-Seq data analysis was performed essentially according to the methods described previously^18^. Briefly, the raw RNA-Seq reads from each library were first aligned to a ribosomal RNA database to remove those that were aligned. The cleaned reads were aligned to the melon genome^61^ and an average of ~84% of cleaned reads was mapped to the melon genome. For each melon gene, its raw counts were calculated and normalized to RPKM. The raw counts of melon genes at each of the four developmental stages were analyzed to identify DEGs between CEZ and *low-β* or *yofi* with parameters of greater than or equal to twofold changes and the adjusted *p* values less than 0.05.

### Gene functional classification and enrichment analysis

The DEGs were functionally classified using MapMan^25^. Venn Diagrams were generated using an online tool (http://bioinfogp.cnb.csic.es/tools/venny/index.html). Enrichment analysis was done using Gorilla (http://cbl-gorilla.cs.technion.ac.il/) and gene clustering to functional groups was done according to MapMan hierarchical classification (http://mapman.gabipd.org/web/guest/pageman).

### RT-qPCR analysis

Total RNA from samples of three CEZ and three *low-β* mature fruits, which were used for RNA-Seq analysis, was used here. The cDNA was synthesized using 1 µg of RNA, 2.5 μl of 20 µM of oligo(dT), 2 μl of dNTP (10 mM each), and the MMLV Reverse Transcriptase (GPR Kit, Takara). The RT-qPCR mixture comprised of 5 μl of iQ SYBR^®^ Green Supermix (Bio-Rad), 0.5 μl of each primer (10 μM), 1 μl of cDNA (5x diluted), and 3 μl of distilled water. The reactions using gene specific primers (Table S8) were performed on a CFX384 Touch Real-Time PCR Detection System (Bio-Rad), with three technical repeats per sample and water control for each primer pair. The PCR was carried out with a reaction initiation at 95 °C for 3 min, followed by 40 cycles of 95 °C for 10 s and 55 °C for 30 s. Melting curves were constructed by gradual heating from 55 to 95 °C, with an increase of 0.5 °C and 5 s holding time per step. Expression levels of the target genes were normalized against Actin and calculated using the 2^−ΔΔ^ CT method. Correlation analysis between RNA-Seq and RT-qPCR expression data was performed with cor.test in R software (https://cran.r-project.org).

### BiFC assay

To make the BiFC constructs, the CmOR (MELO3C005449), CmOR truncated form^19^, and CmOR-like (MELO3C024554) CDS without stop codon were amplified using primers with appropriate restriction enzyme sequences added (Table S8), cloned into PCR2.1 (Invitrogen), and subcloned into pSPYNE173 vector through XbaI and XhoI sites to produce CmOR-nYFP or ORL-nYFP fusion protein constructs. The two genes *MELO3C007154* and *MELO3C012912* encoding LHCB1.1 and LHCB1.5 protein, respectively, were cloned to pSPYCE(M) through XbaI and XhoI sites to produce cYFP fusion protein constructs. These constructs were transformed into *Agrobacterium tumefaciens* strain GV3101. Pairs of nYFP and cYFP fusion plasmids were infiltrated into tobacco (*Nicotiana benthamiana*) leaves. Two days after infiltration, the tobacco leaf samples were observed under a laser confocal microscope (Leica TCS SP5 Laser Scanning Confocal Microscope). The YFP fluorescent signals were detected with excitation wavelength at 514 nm and emission filter between 520 and 560 nm as described previously^35^.

### Y2H analysis

Y2H analysis was performed using the split ubiquitin system as described previously^51^. The *MELO3C007154* (*LHCB1.1*) and *MELO3C012912* (*LHCB1.5*) CDS without the sequences encoding their transit peptides and stop codon were amplified using primers (Table S8) and cloned into Nub vector. The *CmOR* (*MELO3C005449*) and *CmOR-like (MELO3C024554*) CDS were amplified and cloned into Cub vector. The Nub and Cub plasmids were transformed into yeast strain THY.AP4 and THY.AP5, respectively, and mated in pairs. Yeast strains were plated out with a series of tenfold dilutions on non-selection medium and selection medium lacking leucine, tryptophan, and histidine (-LWH) with 5 mM 3-AT to suppress the background growth.

Quantification of the interaction strength by the β-galactosidase activity assay was carried out as detailed^62^. Briefly, five clones from each yeast pairs were inoculated and grew overnight at 30 °C in 5 ml of SD/LW liquid medium. The activity was measured in a reaction mixture with o-nitrophenyl-β-galactoside as substrate and expressed as Miller units^62^.

## Supplementary information

Table S1

Table S2

Table S3

Table S4

Table S5

Table S6

Table S7

Table S8

## Data Availability

The raw RNA-Seq reads have been deposited in the National Center for Biotechnology Information BioProject database (http://www.ncbi.nlm.nih.gov/bioproject) with ID PRJNA690191.

## References

[1] Nisar N, Li L, Lu S, Khin NC, Pogson BJ (2015). Carotenoid metabolism in plants. Mol. Plant.

[2] Rodriguez-Concepcion M (2018). A global perspective on carotenoids: metabolism, biotechnology, and benefits for nutrition and health. Prog. Lipid Res..

[3] Sun T, Li L (2020). Toward the ‘golden’ era: the status in uncovering the regulatory control of carotenoid accumulation in plants. Plant Sci..

[4] Ramel F (2012). Chemical quenching of singlet oxygen by carotenoids in plants. Plant Physiol..

[5] Jia K-P, Baz L, Al-Babili S (2018). From carotenoids to strigolactones. J. Exp. Bot..

[6] Finkelstein, R. Abscisic acid synthesis and response. *Arabidopsis Book* **11** (2013). 10.1199/TAB.0166. 10.1199/tab.0166 PMC3833200 24273463

[7] Yuan H, Zhang J, Nageswaran D, Li L (2015). Carotenoid metabolism and regulation in horticultural crops. Hortic. Res..

[8] Hermanns AS, Zhou X, Xu Q, Tadmor Y, Li L (2020). Carotenoid pigment accumulation in horticultural plants. Hortic. Plant J..

[9] Lewinsohn E (2005). Not just colors—carotenoid degradation as a link between pigmentation and aroma in tomato and watermelon fruit. Trends Food Sci. Technol..

[10] Ibdah M (2006). Functional characterization of CmCCD1, a carotenoid cleavage dioxygenase from melon. Phytochemistry.

[11] Sun T (2018). Carotenoid metabolism in plants: the role of plastids. Mol. Plant..

[12] Zhou X (2015). Arabidopsis OR proteins are the major posttranscriptional regulators of phytoene synthase in controlling carotenoid biosynthesis. Proc. Natl Acad. Sci. USA.

[13] Isaacson T, Ronen G, Zamir D, Hirschberg J (2002). Cloning of tangerine from tomato reveals a carotenoid isomerase essential for the production of β-carotene and xanthophylls in plants. Plant Cell.

[14] Park H, Kreunen SS, Cuttriss AJ, DellaPenna D, Pogson BJ (2002). Identification of the carotenoid isomerase provides insight into carotenoid biosynthesis, prolamellar body formation, and photomorphogenesis. Plant Cell.

[15] Sun, T., Tadmor, Y. & Li, L. *Pathways for Carotenoid Biosynthesis, Degradation, and Storage* 3–23 (Humana, 2020). 10.1007/978-1-4939-9952-1_1 31745909

[16] Burger Y (2013). Genetic variability for valuable fruit quality traits in *Cucumis melo*. Isr. J. Plant Sci..

[17] Tzuri G (2015). A “golden” SNP in *CmOr* governs the fruit flesh color of melon (*Cucumis melo*). Plant J..

[18] Chayut N (2015). A bulk segregant transcriptome analysis reveals metabolic and cellular processes associated with *Orange* allelic variation and fruit β-carotene accumulation in melon fruit. BMC Plant Biol..

[19] Chayut, N. et al. Distinct mechanisms of the ORANGE protein in controlling carotenoid flux. *Plant Physiol*. **173** (2017). 10.1104/pp.16.01256. 10.1104/pp.16.01256 PMC5210724 27837090

[20] Feder A (2019). The role of carotenogenic metabolic flux in carotenoid accumulation and chromoplast differentiation: lessons from the melon fruit. Front. Plant Sci..

[21] Tadmor Y (2007). Induced mutagenesis to augment the natural genetic variability of melon (*Cucumis melo* L.). Isr. J. Plant Sci..

[22] Galpaz N (2013). Genetic and chemical characterization of an EMS induced mutation in *Cucumis melo* CRTISO gene. Arch. Biochem. Biophys..

[23] Jin B (2019). Analysis of flesh color-related carotenoids and development of a CRTISO gene-based DNA marker for prolycopene accumulation in watermelon. Hortic. Environ. Biotechnol..

[24] Zhang J (2015). Molecular characterization and transcriptome analysis of orange head Chinese cabbage (*Brassica rapa* L. ssp. pekinensis). Planta.

[25] Thimm O (2004). mapman: a user-driven tool to display genomics data sets onto diagrams of metabolic pathways and other biological processes. Plant J..

[26] Osorio CE (2019). The role of orange gene in carotenoid accumulation: manipulating chromoplasts toward a colored future. Front. Plant Sci..

[27] Brogna S, Wen J (2009). Nonsense-mediated mRNA decay (NMD) mechanisms. Nat. Struct. Mol. Biol..

[28] Kachanovsky DE, Filler S, Isaacson T, Hirschberg J (2012). Epistasis in tomato color mutations involves regulation of phytoene synthase 1 expression by cis-carotenoids. Proc. Natl Acad. Sci. USA.

[29] Chai C (2011). ZEBRA2, encoding a carotenoid isomerase, is involved in photoprotection in rice. Plant Mol. Biol..

[30] Fantini E, Falcone G, Frusciante S, Giliberto L, Giuliano G (2013). Dissection of tomato lycopene biosynthesis through virus-induced gene silencing. Plant Physiol..

[31] Álvarez D (2016). Carotenogenesis is regulated by 5′utr-mediated translation of phytoene synthase splice variants. Plant Physiol..

[32] Avendaño-Vázquez A-O (2014). An uncharacterized apocarotenoid-derived signal generated in ζ-carotene desaturase mutants regulates leaf development and the expression of chloroplast and nuclear genes in *Arabidopsis*. Plant Cell.

[33] Cazzonelli CI (2020). A cis-carotene derived apocarotenoid regulates etioplast and chloroplast development. Elife.

[34] Alagoz Y, Nayak P, Dhami N, Cazzonelli CI (2018). cis-carotene biosynthesis, evolution and regulation in plants: the emergence of novel signaling metabolites. Arch. Biochem. Biophys..

[35] Yuan H (2015). A single amino acid substitution in an orange protein promotes carotenoid overaccumulation in *Arabidopsis*. Plant Physiol..

[36] Li L (2006). β-carotene accumulation induced by the cauliflower *Or* gene is not due to an increased capacity of biosynthesis. Phytochemistry.

[37] Stanley L, Yuan Y-W (2019). Transcriptional regulation of carotenoid biosynthesis in plants: so many regulators, so little consensus. Front. Plant Sci..

[38] Sun T (2019). ORANGE represses chloroplast biogenesis in etiolated *Arabidopsis* cotyledons via interaction with TCP14. Plant Cell.

[39] Cazzonelli CI, Yin K, Pogson BJ (2009). Potential implications for epigenetic regulation of carotenoid biosynthesis during root and shoot development. Plant Signal Behav..

[40] Llorente B (2020). Synthetic conversion of leaf chloroplasts into carotenoid-rich plastids reveals mechanistic basis of natural chromoplast development. Proc. Natl Acad. Sci. USA.

[41] Hok S (2014). The receptor kinase impaired oomycete susceptibility1 attenuates abscisic acid responses in *Arabidopsis*. Plant Physiol..

[42] Mochizuki N (2010). The cell biology of tetrapyrroles: a life and death struggle. Trends Plant Sci..

[43] Ayub R (1996). Expression of ACC oxidase antisense gene inhibits ripening of cantaloupe melon fruits. Nat. Biotechnol..

[44] John I (1995). Delayed leaf senescence in ethylene-deficient ACC-oxidase antisense tomato plants: molecular and physiological analysis. Plant J..

[45] Joshi, R., Ramanarao, M. V. & Baisakh, N. *Arabidopsis* plants constitutively overexpressing a myo-inositol 1-phosphate synthase gene (SaINO1) from the halophyte smooth cordgrass exhibits enhanced level of tolerance to salt stress. *Plant Physiol. Biochem*. **65**, 61–66 (2013). 10.1016/j.plaphy.2013.01.009 23416497

[46] Falk S, Sinning I (2010). cpSRP43 is a novel chaperone specific for light-harvesting chlorophyll a,b-binding proteins. J. Biol. Chem..

[47] Eden E, Navon R, Steinfeld I, Lipson D, Yakhini Z (2009). GOrilla: a tool for discovery and visualization of enriched GO terms in ranked gene lists. BMC Bioinform..

[48] Ahrazem O (2020). Differential interaction of Or proteins with the PSY enzymes in saffron. Sci. Rep..

[49] Wang Z (2015). Transgenic Alfalfa plants expressing the sweetpotato orange gene exhibit enhanced abiotic stress tolerance. PLoS ONE.

[50] Park S (2016). Orange protein has a role in phytoene synthase stabilization in sweetpotato. Sci. Rep..

[51] Welsch R (2018). Clp protease and OR directly control the proteostasis of phytoene synthase, the crucial enzyme for carotenoid biosynthesis in *Arabidopsis*. Mol. Plant.

[52] Welsch R (2020). Characterization of cauliflower OR mutant variants. Front. Plant Sci..

[53] Sun T (2020). OR^His^, a natural variant of OR, specifically interacts with plastid division factor ARC3 to regulate chromoplast number and carotenoid accumulation. Mol. Plant.

[54] Kim HS (2018). Orange: a target gene for regulating carotenoid homeostasis and increasing plant tolerance to environmental stress in marginal lands. J. Exp. Bot..

[55] Pulido P, Leister D (2018). Novel DNAJ-related proteins in *Arabidopsis thaliana*. N. Phytol..

[56] Jansson S (1999). A guide to the Lhc genes and their relatives in *Arabidopsis*. Trends Plant Sci..

[57] Humbeck K, Krupinska K (2003). The abundance of minor chlorophyll a/b-binding proteins CP29 and LCHI of barley (*Hordeum vulgare* L.) during leaf senescence is controlled by light. J. Exp. Bot..

[58] Liu R (2013). Light-harvesting chlorophyll a/b-binding proteins, positively involved in abscisic acid signalling, require a transcription repressor, WRKY40, to balance their function. J. Exp. Bot..

[59] Staneloni RJ, Rodriguez-Batiller MJ, Casal JJ (2008). Abscisic acid, high-light, and oxidative stress down-regulate a photosynthetic gene via a promoter motif not involved in phytochrome-mediated transcriptional regulation. Mol. Plant.

[60] Kang L (2017). IbOr regulates photosynthesis under heat stress by stabilizing ibpsbp in sweetpotato. Front. Plant Sci..

[61] Garcia-Mas J (2012). The genome of melon (*Cucumis melo* L.). Proc. Natl Acad. Sci. USA.

[62] Yuan H (2021). Arabidopsis ORANGE protein regulates plastid pre-protein import through interacting with Tic proteins. J. Exp. Bot..

